# Neutrophil/lymphocyte ratio: a potential biomarker in patients with head and neck squamous cell carcinoma treated with immune checkpoint inhibitors

**DOI:** 10.3389/fonc.2025.1557652

**Published:** 2025-05-06

**Authors:** Marianna Caterino, Giorgio Lo Giudice, Vincenzo Damiano, Francesco Perri, Guido Giordano, Davide Ciardiello, Aurora Mirabile, Mario Pirozzi, Andrea Pietro Sponghini, Vincenzo Ricci, Liliana Montella, Raffaele Addeo, Francesca Vignani, Vincenzo Famiglietti, Stefano Farese, Sara Di Lorenzo, Fortunato Ciardiello, Morena Fasano

**Affiliations:** ^1^ Oncology Unit, Casa di Cura Villa Salus, Messina, Italy; ^2^ Department of Medicine and Surgery, University of Enna “Kore”, Enna, Italy; ^3^ Clinical Department of Oncology and Hematology, University of Naples “Federico II”, Naples, Italy; ^4^ Istituto Nazionale Tumori Di Napoli, IRCCS “G. Pascale”, Naples, Italy; ^5^ Department of Medical and Surgical Sciences, University of Foggia, Foggia, Italy; ^6^ Division of Gastrointestinal Medical Oncology and Neuroendocrine Tumors, European Institute of Oncology (IEO), IRCCS, Milan, Italy; ^7^ Department Unit of Oncology, Medical Oncology Department, IRCCS San Raffaele Scientific Institute, Università Vita-Salute, Milano, Italy; ^8^ SCDU Oncologia, Ospedale Maggiore della Carità, Novara, Italy; ^9^ Medical Oncology Unit, AORN “San Pio”, Benevento, Italy; ^10^ Oncology Operative Unit, “Santa Maria delle Grazie” Hospital, ASL Napoli 2 NORD, Pozzuoli, Italy; ^11^ Oncology Division, Ospedale “San Giovanni di Dio”, Frattamaggiore, Italy; ^12^ Medical Oncology, Mauriziano Hospital, Turin, Italy; ^13^ Precision Medicine Department, Medical Oncology, University of Campania “Luigi Vanvitelli”, Naples, Italy

**Keywords:** HNSCC, NLR, immunotherapy, immunotherapy biomarkers, ICI

## Abstract

**Introduction:**

Literature has shown that there is a correlation between increased circulatory inflammatory factors and negative prognosis, which can be evaluated through the using the neutrophil and lymphocyte ratio (NLR). The aim of this research is to investigate the predictive and prognostic role of the NLR in recurrent/metastatic head and neck squamous cell carcinoma (R/M HNSCC) patients, treated with immunotherapy, and its correlation to the overall survival (OS), progression free survival (PFS) and objective response rate (ORR).

**Methods:**

This multicentric study coordinated by the Oncology Unit of University of Campania “Luigi Vanvitelli”, retrospectively analyzed data from 135 patients diagnosed with R/M HNSCC from 13 Italian oncological centers.

**Results:**

Two groups were made using the median NLR value of 4.2. 71 patients (52.6%) had NLR>4 and 64 patients (47.4%) had NLR<=4. Mean OS of patients with NLR>4 was significantly shorter than that of patients with NLR<=4 (23.1 vs 37.4 months, p= 0.002). Univariable analysis showed a statistically significant correlation between OS and NLR value (p=0.002), and between OS and ECOG (p=0.022). Median PFS stratified by NLR value, was statistically significant: 6.5 vs 20 months in patients with NLR>4 and NLR<=4, respectively (p= 0.013O). ORR in the general population was 32.6%. NLR-stratified ORR confirmed the unfavorable prognostic role of high NLR: 20% if NLR<=4, and 12.5% if NLR>4.

**Discussion:**

Basal NLR value lower than the cut-off of 4 is independently associated with better OS, PFS and ORR in patients with R/M HNSCC treated with immunotherapy, in first- or second- line.

## 1 Introduction

Squamous cell carcinoma of the head/neck cancer (HNSCC) represents a malignant tumor of epithelial origin characterized by a marked chemo-radiosensitivity, which makes it treatable when diagnosed. However, even in cases where it is possible to treat the disease with curative intent (surgery associated adjuvant radiotherapy – RT– with chemotherapy -CT-, or chemoradiotherapy treatment with definitive intent) the risk of locoregional recurrence and/or distant metastasis remains high.

The advent of immunotherapy in clinical practice has revolutionized the therapeutic possibilities of oncology, offering a new therapeutic chance in the treatment of several neoplasms, including those of the head and neck district (1–3). Until the approval in Italy in 2020 of pembrolizumab use, the first-line therapy used in recurrent/metastatic (R/M) HNSCC cases, was the EXTREME protocol (anti-EGFR cetuximab, platinum and 5-fluorouracil). Now in these cases, specifically, pembrolizumab may be used in the forefront, alone or in combination with platinum-based chemotherapy and 5-fluorouracil, in those patients whose tumors express programmed death-ligand 1 (PD-L1) with a combined positive score (CPS) equal to or more than 1, and nivolumab in patients relapsed within six months of treatment with platinum salts or in second line, regardless of the CPS (1, 4). Among the prognostic and predictive factors of head and neck cancer we can include: age, race, performance status (PS) at diagnosis, smoking habit, the stage of neoplasm, a previous RT in the adjuvant/definitive setting, positivity for HPV infection (1).

With the advent of precision medicine, it represents an extremely tempting challenge to search for new prognostic and predictive markers to personalize treatments more and more, thus identifying responders from non-responders, to save patients from the toxicity of unnecessary treatments (5, 6). It is now known that there is a correlation between tumor development/progression and the state of systemic inflammatory response (7). Several studies have shown that there is a correlation between increased circulatory inflammatory factors, which can be evaluated through the ratio of C-reactive protein to albumin, (so-called Glasgow prognostic score) and a negative prognosis (8). Similar results were also observed using the neutrophil to lymphocyte ratio (NLR) (9–12). In fact, while neutrophil appears to be a snapshot of a pro-inflammatory systemic immune response, lymphopenia appears to adversely affect response to immunotherapy treatments. The functioning of programmed cell death protein 1 inhibitor (PD1), in fact, is strictly dependent on the activity of T cells (13). The presence of a predominantly lymphocytic infiltrate pattern, consisting of CD8+ cytotoxic T cells, CD4+ T helper 1 (Th1) cells, Natural Killer (NK) cells, and mature dendritic cells, can induce greater benefits from immunotherapy treatment compared to tumors lacking such infiltrating lymphocytes. Inflamed tumors, therefore, host a large number of T lymphocytes at the periphery and within the tumor, with increased expression of T cell activation markers, type 1 interferon, and high levels of Th1 cytokines and chemokines, which in turn can promote T cell recruitment and activate effector functions. Conversely, the non-inflamed tumor microenvironment has few effector T cells and a prevalence of cells expressing chronic inflammation markers such as macrophages, myeloid-derived suppressor cells (MDSCs), Th2 cytokines, and tumor-associated chemokines, resulting in the creation of an immunosuppressed microenvironment that allows tumor progression. Therefore, understanding whether a tumor is immunologically hot or cold has important therapeutic implications. In immunologically hot tumors, the problem is that cancer acts by activating molecular brakes (e.g., the PD-1/PD-L1 inhibitory signaling axis) that act on immune cells. The use of checkpoint antibodies that deactivate this brake, acting on the function of PD-1/PD-L1, allows T cells to regain their activity as tumor-recognizing cells (in practice, the “block” on immune activation is “unblocked”) (14–16).

With our study we wanted to investigate the predictive and prognostic role of the neutrophil/lymphocyte relationship in patients with R/M HNSCC, treated with immunotherapy (in first- or second-line) assessing the correlation between the value of NLR and the overall survival (OS), the progression free survival (PFS) and the objective response rate (ORR) of the patient population.

## 2 Materials and methods

The department of Oncology of the University of Campania “Luigi Vanvitelli” coordinated the retrospective collection of data of patients diagnosed with HNSCC, from 13 Italian oncological centers (A.O.U. “Luigi Vanvitelli”, Napoli; A.O.U. “Federico II”, Napoli; IRCSS Pascale, Napoli; A.O.U. Ospedali Riuniti, Foggia; A.O.R.N. Sant’Anna e San Sebastiano, Caserta; Casa Sollievo della Sofferenza, San Giovanni Rotondo; IRCCS San Raffaele, Milano; Ospedale Maggiore della Carità, Novara; A.O.R.N. “San Pio”, Benevento; Ospedale Santa Maria delle Grazie, Pozzuoli; Ospedale “San Giovanni di Dio”, Frattamaggiore; Ospedale Mauriziano, Torino; Casa di Cura Villa Salus, Messina).

We included patients older than 18, with histological diagnosis of HNSCC, performance status (PS) according to Eastern Cooperative Oncology Group (ECOG) between 0-2, who had received immunotherapy in the first- or second- line of treatment, and whose pre-treatment NLR values were known. Data were censored on 31/01/2024. The study was approved by the local ethics committee (prot. N°280, 06/10/2020).

### 2.1 Patient population

Data from 142 HNSCC patients were collected retrospectively from 2016 to 2024. Of these 142 patients 5 were excluded because they did not undergo immunotherapy treatment either in first- or second- line, 2 were excluded due to lack of data. 135 patients were included in the final analysis.

### 2.2 Data

We collected retrospectively patient data, including age, sex, body mass index (BMI), ECOG PS, smoking and/or alcohol habit, primary tumor localization, histology, stage, co-morbidity, previous treatments (including surgery, RT and/or adjuvant systemic medical therapy), treatments used in first- and second- line, site of re-appearance of disease and/or metastasization, NLR value, response and duration of treatment response.

The BMI was calculated by dividing the weight in kilograms by the square height in square meters, and a value of 18 was threshold for underweight. The NLR was calculated by dividing the neutrophil count by the lymphocyte count (obtained by researching in the blood registers of the different hospitals involved) before therapy. Stage was defined using the American Joint Commission on Cancer (AJCC) 8^th^ edition classification. Response to treatment was measured by each center involved independently using Response assessment Evaluation Criteria In Solid Tumors (RECIST) version 1.1.

To analyze the difference in outcomes, both OS and PFS stratified according to the pre-treatment value of NLR, we divided the patients, using the median NLR value of 4.2, into two groups: patients with NLR <= 4 and NLR > 4.

### 2.3 Objectives

Primary endpoint is the stratified OS based on the value of NLR<=4 or > 4; secondary endpoints are the PFS similarly stratified according to the cut off value of the NLR, and the objective response rate (ORR), stratified on the basis of the values of the neutrophil lymphocyte ratio.

### 2.4 Statistical analysis

Statistical analysis was performed with the software “Statistical Package for Social Sciences” (SPSS). The statistical significance of all hypothesis tests (α) was set to 0.05, so that a p < 0.05 indicated a statistically significant difference.

OS was calculated from the start date of immunotherapy (first- or second- line) and death for any cause; PFS was evaluated from the date of onset of immunotherapy (first- or second- line) and the date of disease progression (distant metastases, locoregional recurrence or death for any cause). The median follow-up period was evaluated using the reverse Kaplan Meier. ORR of the general population and the two subgroups of patients were compared. The probability of survival (OS and PFS) was calculated using the Kaplan-Meier method, with a 95% confidence interval (C.I.). The survival curves thus obtained were compared with the Log-Rank Test, in order to evaluate the differences in survival, based on the NLR value. Finally, a univariable and multivariate survival analysis was performed with the Cox proportional hazards regression model. In this case, after having established with univariable analysis the unfavorable prognostic role correlated with a high NLR value, all known prognostic variables (immunotherapy administration setting, BMI, smoking habit and previous RT) were included in the multivariate analysis to confirm the independent prognostic value of the NLR.

## 3 Results

### 3.1 Patient population

135 patients were included in the study, stratified on the basis of NLR in two groups. All patients had squamous cell carcinoma. The median of NLR was 4.2 (range 1 - 34), with 64 patients (47.4%) in the group with NLR<=4 and 71 patients (52.6%) with NLR >4. Basal characteristics of the population are reported in **Tables 1**, **2**.

**Table 1:** Patients’ demographics.

Total Patients: 135			
**Age**		Mean	62
		Min	34
		Max	90
	N, (%)	≤60	56 (41.5)
		>60	79 (58.5)
**Sex**	N, (%)	Female	43 (31.8)
		Male	92 (68.2)
**ECOG**	N, (%)	0	60 (44.5)
		1	62 (45.9)
		2	13 (9.6)
**Smoker**	N, (%)	Yes	112 (83)
		No	23 (17)
**Alcohol use**	N, (%)	Yes	48 (35.6)
		No	87 (64.4)
**BMI**		Median	24
		Min	15
		Max	46
	N, (%)	≤18	14 (10.4%)
		>18	121 (89.6)
**NLR**		Median	4.2
		Min	1
		Max	34
	N, (%)	≤4	64 (47.4)
		>4	71 (52.6)
**Tumor Localization**	N, (%)	Nasopharynx	2 (1.5)
		Oropharynx	14 (10.4)
		Tonsil	7 (5.2)
		Hypopharynx	7 (5.2)
		Larynx and pharynx	1 (0.7)
		Larynx	55 (40.7)
		Nasal sinus	2 (1.5)
		Oral cavity	43 (31.9)
		Parotid gland	1 (0.7)
		Neck lymph node from occult primitive	3 (2.2)
**Staging**	N, (%)	II-III	58 (42.9)
		IV	77 (57.1)

**Table 2:** Subgroups’ demographics.

			NLR ≤4	NLR >4
**Age**	N, (%)	≤60	27 ()	28
		>60	37 ()	43
**Sex**	N, (%)	Female	23	20
		Male	41	51
**ECOG**	N, (%)	0	34	26
		1	26	36
		2	4	9
**Smoker**	N, (%)	Yes	53	59
		No	11	12
**Alcohol use**	N, (%)	Yes	21	27
		No	43	44
**BMI**	N, (%)	≤18	10	4
		>18	51	62
**Staging**	N, (%)	II-III	29	33
		IV	31	34

75 patients (55.6%) had undergone surgery, and 94 patients (69.6%) had received RT. 84 patients (62.2%) had received systemic medical treatment concurrently with RT (58 (69%) with cisplatin, 5 (6%) with carboplatin and 8 (9.5%) with cetuximab; in 13 patients (15.5%) the data was not found. At the time of progression 62 patients (45.9%) had resumption of non-visceral disease (locoregional, lymph node, bone) and 53 (39.3%) of visceral metastases patients. Specifically, 59 patients had lung involvement, 6 hepatic and 1 cerebral. In 20 patients, disease progression localization was not specified. 85 patients (63%) had received first-line immunotherapy (alone or in association with chemotherapy) and 50 (37%) in second-line. In first-line, 4.4% of patients were treated with CT alone, 23.7% with CT + anti EGFR, 34.1% with combination of platinum-based CT and immunotherapy, 1.5% with single agent anti EGFR, 34.1% with only immune checkpoint inhibitors (ICI); 4 patients were treated in II line with nivolumab.

### 3.2 Response to treatments and prognostic factors

**Figure 1:**
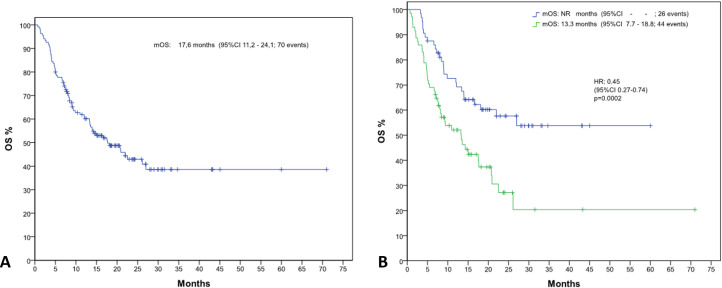
Kaplan-Meier curves depicting overall survival (OS) **(A)** for the entire patient population and **(B)** stratified by neutrophil-to-lymphocyte ratio (NLR) groups (NLR ≤4 and NLR >4).

The average follow-up was 33.8 months for OS and 22.2 months for PFS. At data censoring, 48.1% of patients died, with a median OS of 17.6 months (IC 95%, 11.3-24.1) (**Figure 1A**). Mean OS of patients with NLR >4 was significantly shorter than that of patients with NLR <=4 (23.1 months vs 37.4 months, Hazard Ratio (HR) of 0.45 [IC 95%, 0.27-0.74] p= 0.002) (**Figure 1B**). Median OS in the NLR>4 group was 13.3 months; in the NLR<=4 group the value was not reached.

Univariable analysis showed a statistically significant correlation between the NLR value and OS, p=0.002, and between NLR and ECOG, p=0.022 (**Table 3**). The independent prognostic value of the NLR was confirmed by the multivariate analysis (p=0.005) (**Table 4**).

**Table 3:** Univariate analysis results testing the prognostic role correlated with NLR.

Variables	Univariate analysis	
	HR (95% CI for HR)	p-value
***NLR <=4 vs >4***	0.453 (0.27-0.74)	0.002
***Sex F vs M***	0.66 (0.38-1.14)	0.13
***ECOG 0 vs 1_2***	0.56 (0.34-0.91)	0.022
***Smoking no vs yes***	0.95 (0.51-1.78)	0.88
***Alcohol use no vs yes***	0.90 (0.55-1.48)	0.67
***Age <=60 vs <60***	0.73 (0.45-1.20)	0.22
***BMI >18 vs <=18***	0.73 (0.45-1.20)	0.22
***Stage 4 vs 1_2_3***	0.88 (0.54-1.44)	0.62
***Surgery yes vs no***	0.81 (0.50-1.31)	0.40

**Table 4:** Multivariate analysis results testing the prognostic role correlated with NLR.

Variables	Multivariate analysis	
	HR (95% CI for HR)	p-value
***NLR <=4 vs >4***	0.49 (0.29-0.80)	0.005
***ECOG 0 vs 1_2***	0.62 (0.37-1.029)	0.065

**Figure 2:**
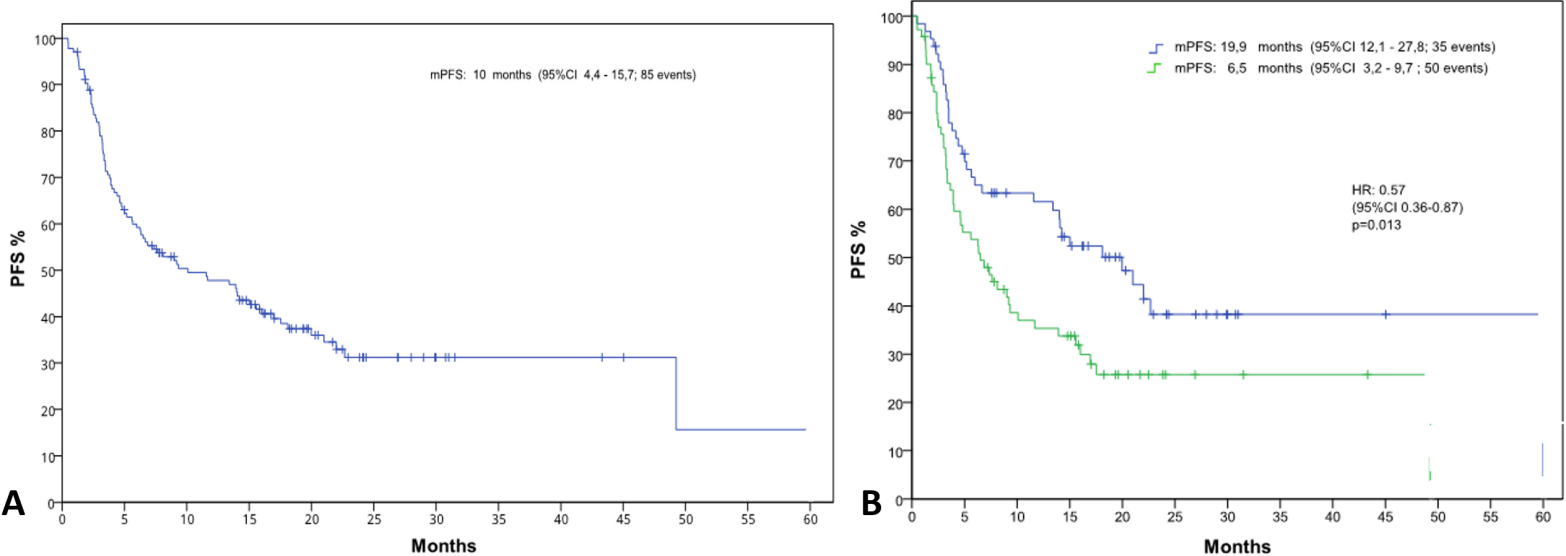
Kaplan-Meier curves illustrating progression-free survival (PFS) **(A)** for the entire patient population and **(B)** stratified by neutrophil-to-lymphocyte ratio (NLR) groups (NLR ≤4 and NLR >4).

The median PFS in the general population was 10.1 months (IC 95%, 4.4-15.8) (**Figure 2A**). Median PFS stratified by the NLR value, was statistically significant: 6.5 vs 20 months in patients with NLR>4 and NLR<=4, respectively (HR 0.57, [IC 95%, 0.36-0.87] p=0.013) (**Figure 2B**).

Finally, we compared the ORR of the general population and the two subgroups of patients. ORR in the general population was 32.6% (out of a total of 135 patients, at the time of censure data were recorded: 42 partial response (PR), 44 stable disease (SD) and 47 progression disease (PD), 2 complete response (CR)). NLR-stratified ORR confirmed the unfavorable prognostic role of high NLR, with better ORR in the subgroup of patients with lower NLR. Specifically, in the subgroup of patients with NLR<=4, ORR was 20% (out of a total of 64 patients: 25 PR, 23 SD, 14 PD and 2 RC); while in the subgroup of patients with NLR>4, ORR was 12.5% (out of a total of 71 patients, 17 PR, 21 SD and 33 PD were registered). PD in the former group was 21.9%, in the latter 46.5%.

## 4 Discussion

Several parameters are routinely evaluated to try to establish the prognosis of a patient with HNSCC, from molecular biomarkers, quality of life assessments, gene expression and serological biomarkers (17–21). Tumor, Node, Metastasis (TNM) stage is considered a standard among prognostic factors of patients with HNSCC. In addition to stage, a low BMI at diagnosis, cigarette smoking habit are associated with a worse prognosis in these patients, while previous RT seems to boost the therapeutic effect of immunotherapy showing better ORR (22, 23). Prognostic factors for assessing the response to immunotherapy in HNSCC have not yet been clearly defined, which is why we have focused our attention on patients suffering from R/M HNSCC who received, in first- or second- line, treatment with anti PD-1 (pembrolizumab alone or in association with chemotherapy based on platinum + 5-fluorouracil and nivolumab) and we analyzed the association between outcomes and basal value of NLR.

The mechanisms behind the complex interaction between high NLR and poor prognosis of cancer patients are still poorly understood. One reason for the prognostic impact of NLR can be found in the association between high levels of NLR and the patient’s inflammatory state. Neutrophils, in fact, release several cytokines that block the host’s immune system against the tumor, thus allowing the neoplasm to grow uncontrollably, suppressing the activity of activated T cells and NK cells (24). Although several points of etiopathogenesis of this interaction have yet to be investigated and understood, the prognostic correlation between a high NLR and a worse prognosis in tumors of different anatomical districts is validated, including the head and neck (25–50). A high NLR is an established negative, independent, prognostic factor, for OS and appears to be related to both stage and PS at diagnosis (51).

Our study found a correlation between NLR<=4 and a better OS of patients treated with immunotherapy, in first- or second- line. The phase III KEYNOTE-048 (KN-048) trial, which compared in the first-line the standard treatment (EXTREME) with the use of pembrolizumab, alone or in combination with platinum-based chemotherapy, showed a median OS in the general population of 14.4 vs 6.5 months in the treated arm with only pembrolizumab and a 16 vs 5.2 months OS in the treated arm with pembrolizumab + CT (52). The OS data in our study cannot be compared with KN-048 since our OS was evaluated in all patients treated with immunotherapy, regardless of the treatment line. The KN-048 study reported the OS stratifying patients on the basis of the treatment received and the CPS value (> 20 and 1). Unfortunately, it was not possible in our case to stratify the OS obtained also on the basis of the value of the PD-L1, measured by CPS, since we did not have this data available in all patients. As for the study of the correlation between high NLR and reduced PFS, our analysis showed a statistically significant difference, with a delta of about 13.4 months in the two groups (19.9 vs 6.5).

Finally, our study showed better response rates in patients treated with immunotherapy and an NLR<=4 (ORR of 20% vs 12.5% respectively in the two groups). Notably, only two complete responses were observed in this group. The phase III CheckMate 141 (CM-141) trial reported an ORR of 13.3% with nivolumab, not comparable with the ORR of our study as it was evaluated in the sample of patients treated with immunotherapy, both at first- than in second- line, and not just in second- line as in the CM-141 study (53).

For our analysis we used a cut off value for NLR of 4 (considered the median of 4.2 in our sample). Several studies have investigated the connection between NLR and prognosis, using NLR cut-off values between 2 and 7 (30, 51, 54–58). Templeton et al. reported that the method used for selecting the NLR cut-off is often unclear, and several authors also demonstrated an association between the NLR cut-off used and the HR values reported for OS (59). Nonetheless, the effect of this association is very little relevant, and it is not clear how it influences the interpretation of the results on the data obtained from the analyses on NLR and OS. Moreover, to establish an absolute cut-off value, a control group of healthy subjects should be employed in such studies. Finally, it cannot be excluded that the optimal cut-off of NLR must be individualized based on the tumor site and the cohort of patients examined, but to determine this we need further investigation.

A limitation of our study is the small number of patients included, with a sample of 135 patients. However, we must correlate the number with the rather rare frequency of the pathology being studied. Further attention should be paid to the short observation time between the start of the first- or second- treatment line and the closure of the observation. Probably subsequent data analysis as well as reconfirming the statistically significant correlation between high NLR and negative prognostic impact in terms of OS could show a statistically significant correlation also between high NLR and shorter NLR PFS. Moreover, the limits related to the type of retrospective analysis, which by its nature can be associated with selection and survival bias.

In addition to the previously highlighted limits, we must consider that in our study neutrophils have not been isolated for further phenotypic characterization. It has recently been discovered that the role played by neutrophils on the microenvironment and on tumor biology varies depending on the predominant phenotype, distinct in pro-tumorigenic and anti-tumor, due to cytokines released into the bloodstream. To further complicate the close correlation between host immune system and tumor development there is also the percentages of the two phenotypes of neutrophils that are not constant over time but, on the contrary, tend to change, a phenomenon known as “Dynamic change of NLR” (56). Since phenotype characterization has not been carried out in our sample, the heterogeneity of pro-tumorigenic and anti-tumorigenic phenotypes cannot be assessed.

Although several studies have demonstrated a prognostic role of dynamic changes in NLR in various cancers, due to data limitations, including missing post-immunotherapy NLR values, incomplete PD-L1/CPS data, and the lack of HPV infection information, we were unable to explore several potentially important associations (50–58, 60–64). Specifically, we could not assess the prognostic role of dynamic NLR changes or stratify OS by PD-L1/CPS since data were often recorded as ranges (e.g., ‘>20’, ‘<1’), and we could not investigate the relationship between NLR and HPV status. Moreover, the small sample size, consisting only of white individuals, underscores the need for research that includes diverse populations to ensure generalizability.

## 5 Conclusions

Our study shows that a basal NLR value lower than the cut-off of 4 is independently associated with better OS, PFS and ORR in patients with R/M HNSCC treated with immunotherapy, in first- or second- line.

Our study presents limitations to be addressed: the small sample considered and the type of retrospective analysis that is, by nature, associated with potential biases, which could limit the validity of the results obtained. Despite the sample size, according to our analysis, NLR is also a useful biomarker in clinical practice, especially considering that it is easy to perform, repeatable, reliable, widely available and inexpensive. Furthermore, we cannot ignore the fact that NLR appears to be directly related to the immune status, inflammation and nutritional status of the host and it is therefore likely that its baseline value reflects the general condition of the patient, and that the better condition of the patient is, in turn, responsible for an impact on the effectiveness of immunotherapy, and therefore, ultimately, also prolonged improvements to the OS. In fact, what is not yet clear is whether the systemic inflammatory response is the reflection of a tumor that does not respond to treatments or a non-specific biomarker of the presence of a systemic proinflammatory state, associated ab initio with reduced survival. If this were the case, then NLR should be assessed in the same way as the correlation between prognosis and PS at diagnosis.

In light of these considerations, further studies are therefore necessary to confirm not only this result, but also to determine the weight of NLR in the context of other biomarkers commonly used for the evaluation of a potential benefit to the use of immunotherapy and above all to evaluate the mutual interaction of different prognostic factors. Only in this way will it be possible to establish in advance which patients will be able to benefit from immunotherapy, so as to be able to improve the prognosis as much as possible of a disease, which is extremely aggressive by its nature.

## Data Availability

The raw data supporting the conclusions of this article will be made available by the authors, without undue reservation.
